# The Role of Hydraulic Failure in a Massive Mangrove Die-Off Event

**DOI:** 10.3389/fpls.2022.822136

**Published:** 2022-04-27

**Authors:** Alice Gauthey, Diana Backes, Jeff Balland, Iftakharul Alam, Damien T. Maher, Lucas A. Cernusak, Norman C. Duke, Belinda E. Medlyn, David T. Tissue, Brendan Choat

**Affiliations:** ^1^Hawkesbury Institute for the Environment, Western Sydney University, Richmond, NSW, Australia; ^2^Plant Ecology Research Laboratory PERL, Ecole Polytechnique Fédérale de Lausanne EPFL, Lausanne, Switzerland; ^3^Swiss Federal Institute for Forest, Snow and Landscape Research WSL, Birmensdorf, Switzerland; ^4^College of Science and Engineering, James Cook University, Cairns, QLD, Australia; ^5^Faculty of Science and Engineering, Southern Cross University, Lismore, NSW, Australia; ^6^TropWATER Centre, James Cook University, Townsville, QLD, Australia

**Keywords:** physiological drought, hydraulic failure, El Niño, *Avicennia marina*, dieback

## Abstract

Between late 2015 and early 2016, more than 7,000 ha of mangrove forest died along the coastline of the Gulf of Carpentaria, in northern Australia. This massive die-off was preceded by a strong 2015/2016 El Niño event, resulting in lower precipitation, a drop in sea level and higher than average temperatures in northern Australia. In this study, we investigated the role of hydraulic failure in the mortality and recovery of the dominant species, *Avicennia marina*, 2 years after the mortality event. We measured predawn water potential (Ψ_pd_) and percent loss of stem hydraulic conductivity (PLC) in surviving individuals across a gradient of impact. We also assessed the vulnerability to drought-induced embolism (Ψ_50_) for the species. Areas with severe canopy dieback had higher native PLC (39%) than minimally impacted areas (6%), suggesting that hydraulic recovery was ongoing. The high resistance of *A. marina* to water-stress-induced embolism (Ψ_50_ = −9.6 MPa), indicates that severe water stress (Ψ_pd_ < −10 MPa) would have been required to cause mortality in this species. Our data indicate that the natural gradient of water-stress enhanced the impact of El Niño, leading to hydraulic failure and mortality in *A. marina* growing on severely impacted (SI) zones. It is likely that lowered sea levels and less frequent inundation by seawater, combined with lower inputs of fresh water, high evaporative demand and high temperatures, led to the development of hyper-salinity and extreme water stress during the 2015/16 summer.

## Introduction

To support photosynthesis, woody plants must extract water from the soil and transport it to the leaves. Water is transported through the xylem conduits under tension and is prone to cavitation, leading to the formation of gas emboli that block the conduits and reduce hydraulic conductivity. As drought intensifies, tension in the xylem rises, increasing the probability of cavitation. This phenomenon can eventually lead to systemic failure of the water transport system and death by hydraulic failure (McDowell et al., 2008; Brodribb and Cochard, 2009). As a result, xylem vulnerability to cavitation, often represented as Ψ_50_ (i.e., water potential at which 50% of the xylem conductivity is lost), is considered to be an important component of drought tolerance in trees and indicative of the capacity of different plant species to survive during extreme drought events (Choat et al., 2012; Choat, 2013).

Many woody ecosystems are already highly vulnerable due to human activities and climate change. In Australia, estuarine ecosystems and coastal wetlands, including mangroves and saltmarshes, are listed as one of the most vulnerable ecosystems (Laurance et al., 2011; Dixon et al., 2016). Their distribution is fragmented and narrow, making them more vulnerable to environmental stress that could lead to local extinctions (Duke et al., 2007; Laurance et al., 2011). Increased temperature and heatwave frequency, as well as reduced rainfall, can raise the vapor pressure deficit (VPD) and contribute to higher rates of plant water loss through transpiration, which may affect survival in this saline environment (Reef and Lovelock, 2015). Reduced rainfall can also limit water availability in groundwater and lead to local droughts, resulting in a decrease in mangrove survival and reduced biomass (Mafi-Gholami et al., 2020).

From a physiological perspective, mangroves are fascinating, with a range of adaptations that allow them to grow in saline, anoxic substrates and cope with frequent tidal inundation (Ball, 1988). An important consequence of growing in a high salinity substrate is having to overcome an enormous osmotic pressure gradient in order to extract water necessary for transpiration and growth. At the salinity of seawater, this pressure exceeds 2.5 MPa, which is physiologically equivalent to growth in extremely dry soils. Leaf water potential in mangroves is therefore consistently low, ranging from −2.7 to −5.7 MPa (Scholander, 1968). To cope with increased salinity, mangroves exclude and filter the salt in sea water. Some species secrete salt via salt glands situated at the leaf level, whereas others filter salt directly at the root level (Scholander, 1968; Tomlinson, 2016). Similarly to species living in xeric environments, mangroves are resistant to water-stress (e.g., Ψ_50_ ranging from −4 MPa to −8.5 MPa) and can tolerate low water potentials without experiencing significant cavitation (Melcher et al., 2001; Jiang et al., 2021). Thus, while mangroves are effectively exposed to continuous and extreme water stress, they form a highly productive ecosystem (Bouillon et al., 2008). However, despite adaptations to growth at low water potentials, mangrove species are still impacted by drought and salinity stress (Ball and Pidsley, 1995; Mafi-Gholami et al., 2020).

Mild or moderate drought events have been reported to reduce photosynthetic capacity of leaves (in *Avicennia germinans*; Sobrado, 1999, 2006); increase salt secretion rates and decrease stomatal conductance (in *Avicennia germinans*; Sobrado, 2002); lower growth rate (*Sonneratia alba*; Krauss et al., 2007); and cause a loss of canopy (*Avicennia marina* and *Rhizophera mucronate*; Mafi-Gholami et al., 2017). While mangrove ecosystems are known for being resilient to short-term drought periods (Galeano et al., 2017; Andrieu et al., 2020; Mafi-Gholami et al., 2020), more extreme drought events have been associated with mass dieback of mangrove vegetation (Duke et al., 2017; Lovelock et al., 2017).

In the tropics, many drought events are related to El Niño Southern Oscillation (ENSO) anomalies, leading to extensive tree mortality (Rice et al., 2004). In North-East Australia, El Niño is responsible for short-term drops in sea level, increased temperatures and reduced rainfall, which play a crucial role in mangrove dieback (Duke et al., 2017; Galeano et al., 2017). During the El Niño event of 2015–2016, northeast Australia experienced persistent and extreme high temperatures as well as severe drought due to an unusually long dry season over the previous year and below average rainfall. These conditions were coincident with a massive dieback event in mangrove forests growing along the Gulf of Carpentaria coastline. Over 1,000 km of coastline was affected, with a total area of more than 7400 ha of mangrove forests impacted by dieback from late 2015 to early 2016 (Duke et al., 2017; Sippo et al., 2018). Another factor playing a role in the dieback was the extreme variation in sea level (20–30 cm lower) caused by weak winds and cool water (Hamlington et al., 2016; Harris et al., 2017). The drop in sea level affected tides, resulting in less inundation of upper areas of mangroves and increased water-stress (Duke et al., 2021). Although it is difficult to attribute direct causality for the dieback event, previous analyses of environmental data suggest that severe moisture deficit derived from high temperatures, low input of freshwater, and less frequent tidal inundation, was likely to be a central factor causing mass mortality of mangroves (Duke et al., 2017, 2021).

Mass dieback of mangroves associated with extreme heatwaves and drought suggests that there are thresholds of water stress that mangroves can survive and that these thresholds may be more frequently encountered with increasing global temperatures and more extreme oscillations in sea level during drought events (Duke et al., 2017, 2021; Abhik et al., 2021). The dramatic El Niño event of 2015/16 provided a unique opportunity to study water-stress-induced mortality in mangrove species and evaluate hydraulic vulnerability and thresholds of mortality. Despite their exposure to physiological drought and salinity stress, very few studies have investigated the vulnerability of xylem to embolism in mangrove species (Sperry et al., 1988; Tyree and Sperry, 1989; Melcher et al., 2001; Ewers et al., 2004) or the role of hydraulic failure in mangrove dieback events. In this study, we focused on the dominant species found in the Gulf of Carpentaria, in north Queensland, *Avicennia marina*, in order to assess its vulnerability to water stress induced mortality. We determined Ψ_50_ using a non-invasive (optical technique) method and provided an estimation of the impact of physiological drought on plants growing along a natural gradient of water-stress. As *A. marina* is a resilient species, we expected to find it to be particularly resistant to physiological drought and that the dieback observed on inland individuals was caused by intense sea level and climatic events.

## Materials and Methods

### Site and Plant Material

**Figure 1:**
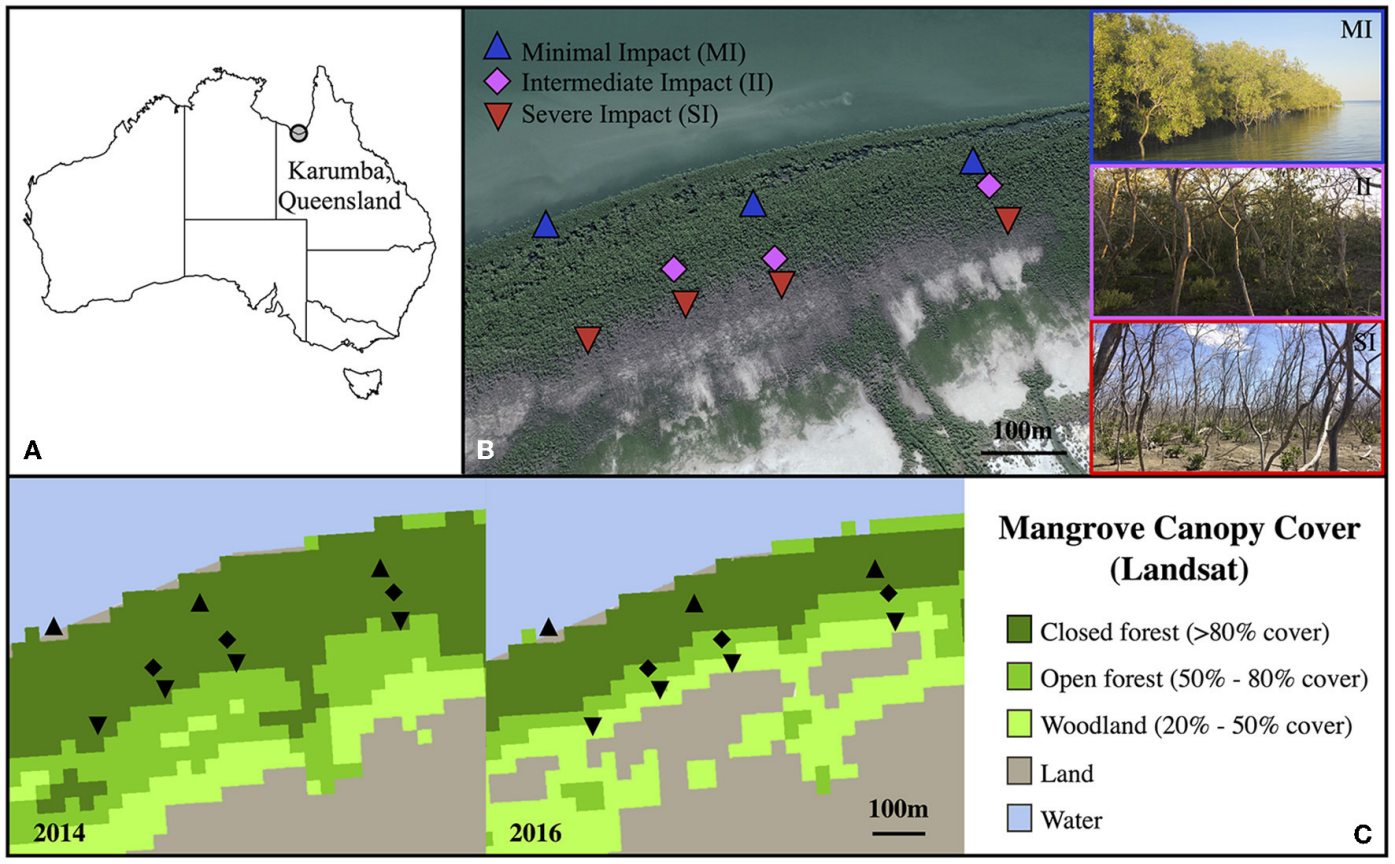
Map showing the field site in Queensland **(A)**, a detail of transects and sampling sites with pictures **(B)** and fractional canopy cover of the field site in 2014 and 2016 **(C)**. In **(B)**, blue triangles represent sites with minimal impact (MI), magenta diamonds sites with intermediate impact (II) and red inverted triangles sites with severe impact (SI). The satellite image was sourced from Google Earth (dated from May 2017). Pictures, taken in August 2018, illustrate the condition of each zones (blue MI, magenta II and red SI). **(C)** Fractional canopy cover (FCC) was assessed using the Digital Earth Australia fractional cover archives Mangrove dataset. Black triangles and lozenges represent the 2018 sampling sites location.

The study site was located in Karumba (Kuthant and Kurtjar countries), in the southern part of the Gulf of Carpentaria (north-west Queensland, Australia; 17° 28'S, 140° 48' E). This site was chosen because of the gradient of tree mortality from seaward to landward zones observed after the 2015–16 dieback event. Trees growing on the landward zone fringing the saltpan, approximately 250 m from the ocean edge, displayed high rates of mortality, while trees growing on the ocean front exhibited no sign of impact and green canopies (Duke et al., 2017; Sippo et al., 2020). While mortality was not explicitly assessed in this study, the impact gradient was clearly visible based on satellite images (Figure 1; mortality data also available from http://wiki.auscover.net.au/wiki/Mangrove) and also assessed by Sippo et al. (2020).

*Avicenna marina* (Forssk.) Vierh. var. *eucalyptifolia* (Zipp. ex Miq.) N. C. Duke, commonly known as the gray mangrove, is one of the most widely distributed mangrove species in the Gulf of Carpentaria, and is globally distributed between 30°N and 38°S (Duke, 1990, 2006). Its latitudinal range covers a wide spectrum of habitats and its growth form is highly plastic, ranging from a shrub (1–3 m) to tall trees (5–10 m) in the tropics. Its secondary growth is achieved by successive cambia. It has lanceolate glossy leaves with salt glands, and aerial roots (pneumatophores), which allow the plant to excrete salt from the sap and absorb oxygen, respectively.

Three transects were established from the saltpan to the oceanfront. Three zones were defined along these transects: severe impact (landward adjacent to saltpan, SI), intermediate impact (II) and minimal impact (seaward fringe, MI) (Figure 1B). The impact severity was directly related, and increased with, the distance of the trees from the waterfront. Trees from MI zone did not show any sign of visible past or present impact whereas trees from SI zone had more than 75% of branches dead or heavily impacted. Trees from II zone had between 25 and 75% of branches dead or impacted.

Fractional canopy cover (FCC) was assessed using the Digital Earth Australia fractional cover archives Mangrove dataset (Figure 1C). FCC estimates were retrieved from Landsat pixels from Landsat 5 (TM), 7 (ETM+), and 8 (OLI), which were decomposed into estimated fractions of photosynthetic vegetation, non-photosynthetic vegetation, and bare ground, using spectral unmixing algorithms developed by the Joint Remote Sensing Research Program (25 m tiles).

All measurements at the site were done in August 2018, two and a half years after the dieback event that occurred in the summer of 2015/2016. During these 2 years between the dieback event and sampling, annual precipitation for this region fluctuated under and above the average of the past 20 years (790 mm; 970 mm in 2017 and 536 mm in 2018), while mean annual temperatures (34°C in 2017 and 33.8°C in 2018) remained above the mean of the past 15 years (33.6°C) (data sourced from Australian Government Bureau of Meteorology, bom.gov.au).

### Native Embolism

Three branches from different trees were collected per zone per transect (i.e., 30 branches total: 1 branch x 3 trees x 3 zones x 3 transect + 3 branches as an additional SI sampling point was added) for native embolism measurements. We harvested branches from adult trees (i.e., >2.5 m tall). In order to assess the impact of past events (i.e., 2015/16 El-Niño), we chose samples that were at least 2 years old. Branches that were collected from SI and II zones had part of their canopy damaged from the previous die-back event but also exhibited regrowth (diameter of ~5 cm). In order to prevent introducing embolism while cutting, we measured the maximum vessel length. This was estimated by applying pressure in the branch, from the basal end and cutting the apical end underwater until bubbles of air were released. The average vessel length was 144 mm and branches over this length could be sampled.

Branches (>1.5 m long) were collected prior to dawn to avoid hydraulic stress associated with the establishment of the transpiration stream occurring after sunrise. Branches were sealed in black plastic bags containing moist paper towels and transported to a temporary laboratory in Karumba. All measurements were undertaken within 2 h after harvesting. One segment of approximately 10 cm long and 5 to 7 mm large per branch was measured (*n* = 30).

Native embolism was assessed by measuring percent loss of conductivity (PLC) of each branch as:


(1) $$\begin{array}{r} PLC=\frac{K_{max}-K_{ini}}{K_{max}}\times 100 \end{array}$$

where *K*_ini_ is the initial hydraulic conductivity and *K*_max_ is the maximum hydraulic conductivity (both, kg s^−1^ m^−1^ MPa^−1^).

Segments of the branches were cut under water and left with the cut end submerged for approximately 20 min in order to release xylem tension and avoid introducing gas bubbles into the segment while cutting (Wheeler et al., 2013). The initial hydraulic conductivity (*K*_ini_) was measured by connecting the sample to a perfusing solution (deionised water and 20 mM NaCl) and attached to a flow meter (LiquiFlow L13-AAD-11-K-10S; Bronkhorst High-Tech B.V., Ruurlo, the Netherlands). The maximum hydraulic conductivity was obtained by measuring the same sample after it was flushed. Flushing was carried out using a syringe filled with degassed perfusing solution that was pressurized using a calking gun (Chapotin et al., 2006). Segments were flushed at a pressure of ~ 100 kPa until *K*_max_ measurements showed no further increase in conductivity (minimum of 20 min).

### Predawn Water Potential

For each native embolism value (i.e., for each branch), predawn stem water potential (Ψ_pd_) was measured and averaged on 2 to 3 leaves. Measurements were conducted using a Scholander pressure chamber (PMS Instrument Company, Albany, OR, USA). Branches were kept in plastic bags for at least 1 h before measurements, to ensure that leaf and stem water potential were equilibrated.

### Vulnerability Curve

Five healthy-looking branches (i.e., full canopy with intact green leaves) were collected for the vulnerability curve (VC). In order to avoid measuring branches with high native embolism that would yield misleading results (i.e., curves shifted toward more negative water potentials), branches were only collected from MI zones. Branches (~1.5 m long) were harvested at predawn from adult trees and measured. The VC was obtained using the optical vulnerability technique (Brodribb et al., 2017). Although there is a possibility that vulnerability to embolism could vary between zones, it was not possible to measure VCs in SI plants because of their severely impacted state at the time of fieldwork. Most recent studies suggest that within species variation of vulnerability to embolism is limited (Lamy et al., 2014; Schuldt et al., 2016; López et al., 2021) and we therefore assume that VCs measured on MI plants are representative of plants across the three zones. If acclimation in VCs was occurring along the transect, it would be expected that SI plants would be more resistant than the VC measured on MI plants (e.g., Bansal et al., 2015).

Segments of 1–2 years old ranging from 0.5 to 1.2 cm in diameter were selected for each branch. A narrow segment was essential in order to see embolism events happening externally and internally in the xylem with OV. A small section of bark was carefully removed to expose the xylem. An 8-megapixel camera with a magnified 20x lens and with light-emitting diodes (LEDs) was placed on the bare xylem and a Raspberry Pi board computer (Raspberry Pi Foundation, http://www.raspberrypi.org) was set to take pictures every 10 minutes. A PSY1 Stem Psychrometer sensor coupled with a microvolt data logger (ICT International, Armidale, NSW, Australia) was used to automatically log measurements of xylem water potential (Ψ_x_) during dry down. The psychrometer was set up on the base of the stem, at least 40 cm apart from the camera in order to minimize the effect of its installation. Bark was removed gently, and the xylem washed with Milli-Q (deionised and filtered water). Once the sensor was installed on the xylem, it was wrapped with parafilm to seal the chamber. Measurements were taken every 10 min to match the camera settings.

Samples were left to dry under plastic bags, in order to limit excessive transpiration, until no embolism events were observed for at least 10 h. Images were downloaded and analyzed using ImageJ (Schneider et al., 2012) following established protocols (https://github.com/OpenSourceOV). After image subtraction, embolism events were clearly revealed, and the area of embolism was calculated for each sample. The percentage of embolism, or percentage of loss of area (PLA), was determined as:


(2) $$\begin{array}{r} \%\;loss\;of\;area=\frac{A_{cav}}{A_{\max}}\;\times \;100 \end{array}$$

where *A*_cav_ is the cumulative area of cavitated xylem at time *t* and *A*_max_ is the maximum area of cavitated xylem at the end of each sample dehydration.

Each embolism event was coupled with water potential measurements, and vulnerability curves were obtained for every sample (*n* = 5).

### Data Analysis

For native embolism, results of PLC and Ψ_pd_ were averaged for each zone (SI, II and MI). After testing for normality, we tested for variation in PLC and Ψ_pd_ among zones with one-way analysis of variance (ANOVA).

Vulnerability curves were fitted to a Weibull function using the *fitplc* package (Duursma and Choat, 2017) with “branch” incorporated as a random effect as the OV technique allows a series of repeated measurements on a single individual. Points of interest (Ψ_12_, Ψ_50_ and Ψ_88_; i.e. water potential corresponding to 12, 50, and 88% of embolism, respectively) and their 95% confidence intervals were extracted from the VC using a standard profiling method.

All statistical analyses were conducted with R 3.1.2 (R Core Team., 2017) using RStudio (RStudio Team., 2015).

## Results

### Conditions of Sites

Comparison of fractional canopy cover (FCC) at the study site between 2014 and 2016 revealed a decrease in FCC that was attributable to the 2015/2016 die off event. While in 2014, all plots were in the highest FCC category (>80 cover), in 2016 there was a reduction in FCC in II (50–80% cover) and SI plots (20% to 50% cover) (Figure 1C).

### Native Embolism

Predawn water potential differed significantly between sites SI (−7.5 MPa ± 0.2), II (−5.6 MPa ± 0.5) and MI (−3.4 MPa ± 0.6) (all *p* < 0.05). While Ψ_pd_ values were highly variable at the MI sites (−0.45 to −5.7 MPa), Ψ_pd_ on the SI sites was less variable (−5.65 to −8.2 MPa) but indicated a high degree of plant water stress.

**Figure 2:**
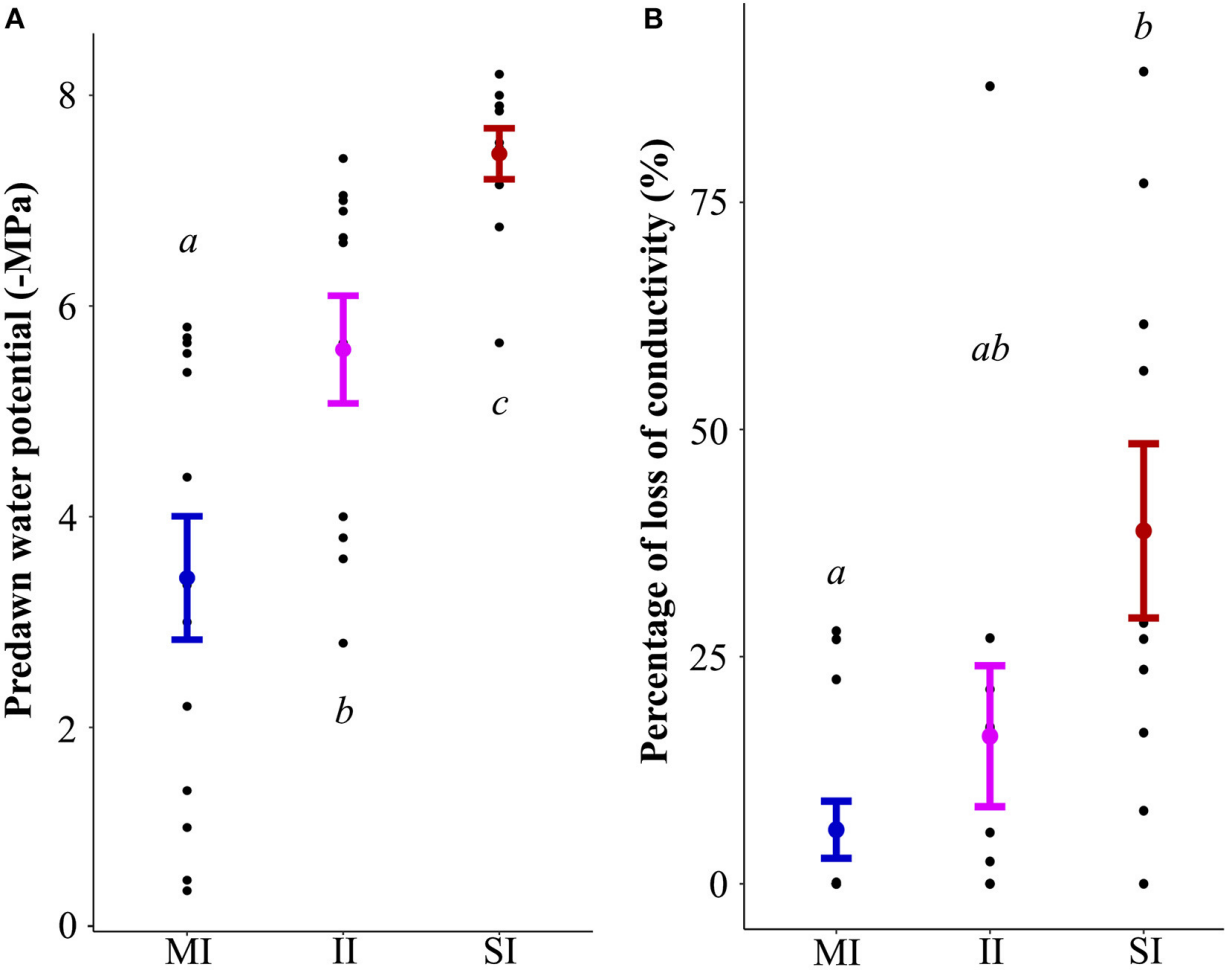
Effect of the degree of impact (MI minimal impact, II intermediate impact and SI severe impact) on predawn water potential (-MPa) **(A)** and percentage loss of conductivity **(B)**. Mean ± standard error. Letters indicate significant differences from the ANOVA (*p* < 0.05) between the sites.

PLC significantly differed between SI (38.8% ± 9.6) and MI sites (5.9% ± 3.1) (*p* = 0.01), but not with II sites (16.2% ± 7.8). On SI sites, PLC values were distributed in a wide range (0 to 89.4%), whereas the distribution of the PLC values on MI sites was narrower (0 to 27.8%) and indicated less hydraulic stress (Figure 2).

### Vulnerability Curves

**Figure 3:**
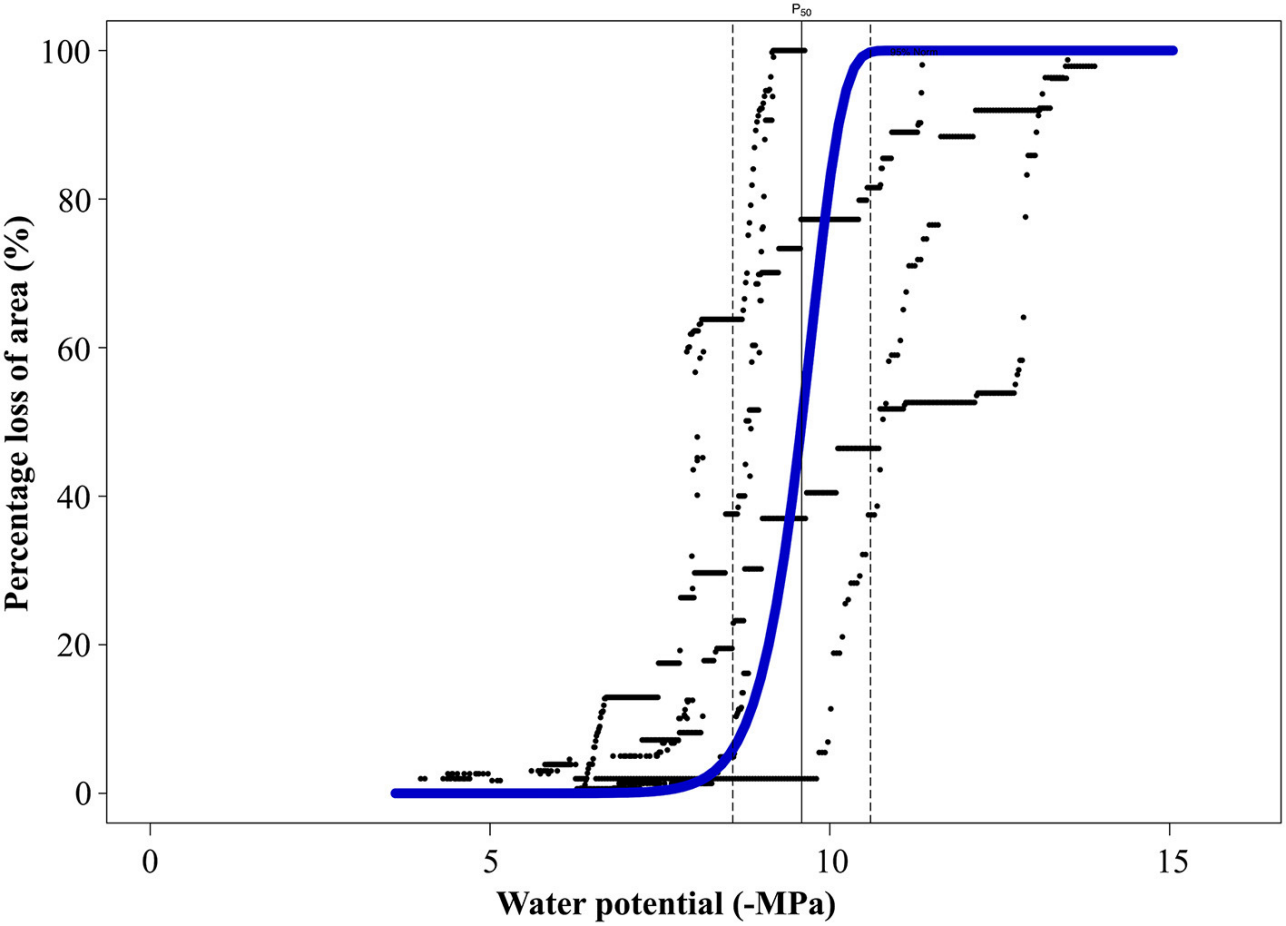
Vulnerability curve (VC) from *A. marina* trees sampled on the sites of minimal impact showing the percentage of loss of area (PLA, %) against the water potential (Ψ_x_, -MPa). The average VC (blue) was built from 5 individual VCs (black). The vertical line shows Ψ_50_ and the dotted lines the 95% confidence intervals for Ψ_50_.

*A. marina* trees from the MI zone exhibited very low vulnerability to embolism (Ψ_12_ −8.87 ± 0.93 MPa; Ψ_50_ −9.59 ± 1.02 MPa; and Ψ_88_ −10.1 ± 1.71 MPa) (Figure 3), indicating a capacity to withstand extremely low water potentials.

**Figure 4:**
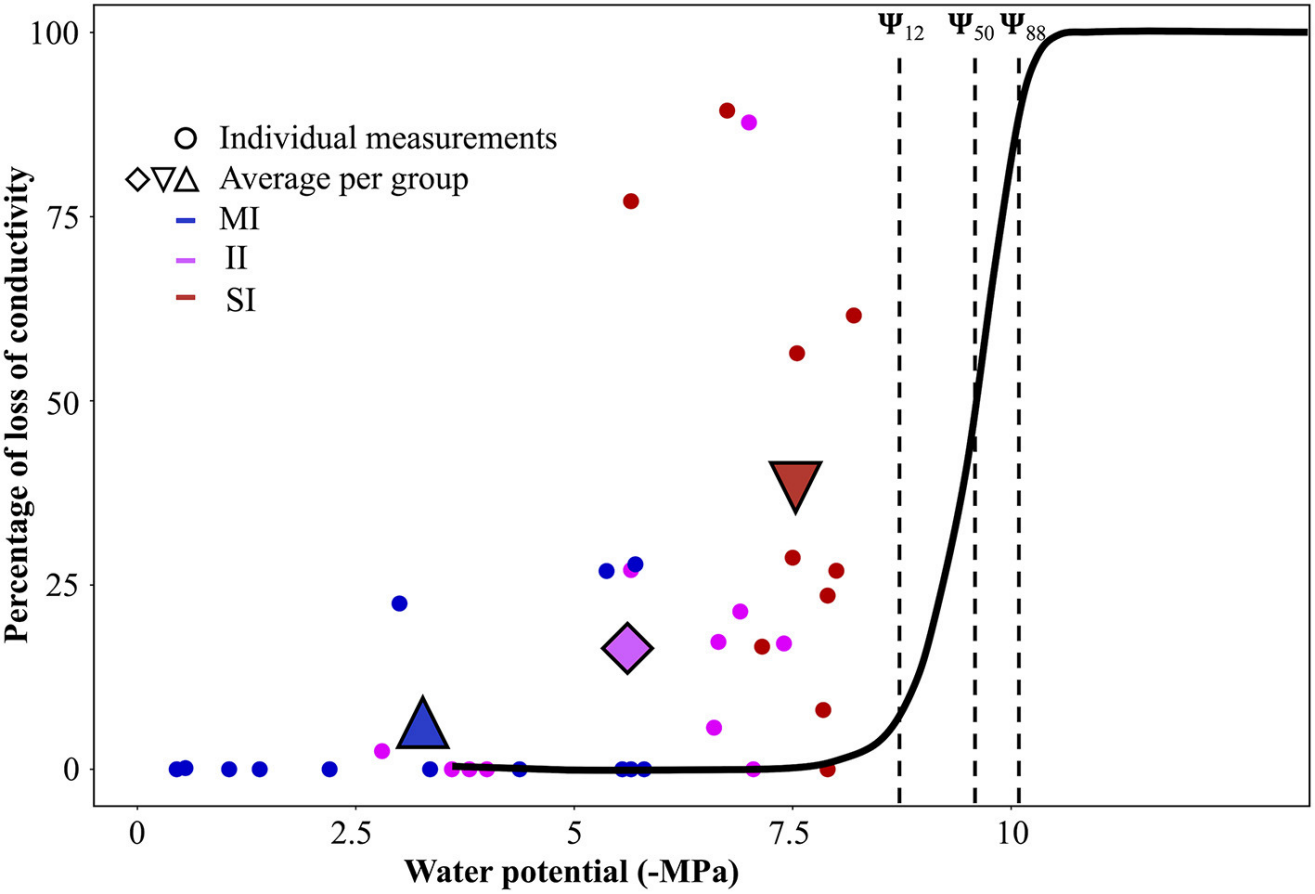
Native embolism measurements with percent loss of conductivity as a function of the water potential in branches sampled at sites of different impact [blue minimal impact (MI), magenta intermediate impact (II), and red severe impact (SI)]. The black sigmoidal curve represents the VC. Points represent an individual measure; triangles and diamonds are the average of these points for each impact class. The dotted lines represent, from left to right, values of Ψ_12_, Ψ_50_, Ψ_88_, extracted from the VC.

Comparison of native embolism and Ψ_pd_ with the vulnerability curve (Figure 4) provided a good opportunity to study hydraulic thresholds that were reached by *A. marina* in each impact zone. Although the average Ψ_pd_ for SI sites was very negative at the time of measurements (Aug. 2018), comparison with the VC showed that mean values of Ψ_pd_ for SI sites (−7.5 MPa ± 0.2) were just below the onset of cavitation (embolism starts at ~−7.1 MPa). Due to intraspecific variation, it should be noted that some individuals used to build the VC began embolising before reaching this water potential (Figure 3); therefore, low Ψ_pd_ on SI sites could potentially cause up to 13% of loss of conductivity in these individuals. The maximum native PLC measured at the time of the experiment (89%) corresponded to Ψ_x_ of −10 MPa on the VC (Figure 4).

## Discussion

We observed high native embolism (PLC) and lower predawn water potentials in surviving trees that had been severely impacted during the dieback event, compared to individuals growing on the seaward fringe (MI). This indicates that trees growing furthest from the seaward fringe were more likely to experience extreme water stress during the die off event because of greater baseline level of water stress of landward mangroves and greater exposure to the effects of the sea level drop observed during the die off event. This is consistent with remotely sensed estimate of FCC, indicating that landward mangroves were the worst impacted populations at this site. After comparison with vulnerability curves, we found that more than 2 years after the die off, individuals growing in SI plots were operating close to their mortality thresholds (Ψ_88_). This indicates that SI individuals were likely predisposed to suffer severe cavitation during a strong El Niño episode and that their distance from the seaward fringe enhanced the risk of water-stress induced mortality. However, given that we were not able to obtain measurements at the site prior to or during the die off event, it is possible that the observed differences in native PLC result from the background variation in water stress across an environmental gradient (seaward to landward). While we cannot eliminate this possibility, the evidence from satellite data suggest that landward zone was differentially impacted by stress during the 2015/16 event. Analyses of climatic processes and sea level anomalies related to the die off event also suggest the imposition of higher levels of plant water stress (Abhik et al., 2021). We therefore speculate that the high PLC measured in SI individuals was caused by intense water-stress that resulted from the combined effects of a strong El-Niño and transient decrease in sea level in the Gulf of Carpentaria.

The strong El Niño event of 2015 resulted in local drops of sea level and high temperatures, leading to increased soil porewater salinity, while lower rainfall resulted in an absence of flushing of the substrate with fresh water and lower groundwater availability (Lovelock et al., 2017; Wang et al., 2020). Moisture derived from rainfall has been shown to specifically influence the extent of mangroves across the tidal profile, where decreases in long-term rainfall result in the expansion of saltmarsh-saltpan zone at the expense of mangrove extent (Duke et al., 2019). While salinity was not measured at our site, the prolonged reduction in sea level can trigger a change from mangrove forests to more saline saltmarsh (Abhik et al., 2021). Moreover, Lovelock et al., 2017 showed that salinity increased in mangrove ecosystems in Western Australia during the same El Niño event. Consequently, mangroves at the Karumba site were likely to have experienced increased salinity, high VPD, and extreme soil water stress.

Significant differences in native embolism in *A. marina* along a transect of water-stress, ~2 years after the ENSO event, suggest that hydraulic failure may have played a role in the dieback in 2015 in the Gulf of Carpentaria. Due to their distance to the waterfront, individuals that were found on the saltpan (SI) were expected to encounter higher water and salinity stress than individuals found on the oceanfront (MI). SI individuals experienced very low water potentials, even prior to dawn, which suggests that some native PLC values measured in this study could have been caused by the daily course of water potential. In *Avicennia*, midday water potential can be between 2 to 5 MPa lower than predawn water potential (Gordon, 1993). However, SI trees were deeply impacted (i.e., many dead branches, low canopy area), thus limiting transpiration rates and the variation of water potential across the day. Moreover, the fractional canopy cover of SI plots was dramatically reduced during the 2015/16 El Niño (see Figure 1C), providing further evidence that the difference in native embolism is unlikely to be explained by the environmental gradient alone. The highest native embolism measured on SI site corresponded to a water potential of −10 MPa (maximum native PLC was 89%) and these PLC values were likely caused by extreme conditions experienced during the die off event rather than by the normal range of water stress. The natural gradient may have accentuated the effect of these events as SI trees were operating closer to cavitation thresholds. In contrast, *A. marina* growing on the oceanfront (MI) was presumably less exposed to the stress caused by the local drop in sea level, which is considered a crucial factor leading the die back event (Abhik et al., 2021).

The Ψ_50_ of *A. marina* was estimated at −9.6 MPa, with no embolism formation detected before −7.1 MPa (from the average VC). Low Ψ_50_ is correlated with high resistance to drought (Blackman et al., 2010; Corcuera et al., 2011; Nardini and Luglio, 2014) and often found in species growing in water limited environments (Nardini and Luglio, 2014; Trueba et al., 2017; Oliveira et al., 2019). Our results are in agreement with previous studies that show that, among plant species, mangroves are quite resistant to drought-induced embolism (Sperry et al., 1988; Choat et al., 2012; Jiang et al., 2017; Zhu et al., 2018). Although *A. marina* was highly resistant to drought-induced embolism, the vulnerability curve exhibited a very steep slope after Ψ_x_ reached Ψ_50_. The difference between Ψ_50_ and Ψ_88_ (0.51 MPa) was smaller than the difference between Ψ_50_ and Ψ_12_ (0.72 MPa), indicating that the rate of embolism spread was higher after the 50% embolism threshold was exceeded.

The comparison of native embolism measurements with the vulnerability curve provided insights regarding the thresholds reached during the 2015–16 El Niño. In order to find this threshold of survival, we focused on branches where native embolism was highest (over 75%, *n* = 3). As previously mentioned, such high native embolism measured in SI sites was most likely caused by hydraulic failure from a past extreme stress event, although we cannot eliminate the possibility that higher native PLC was caused by background levels of stress occurring in their location adjacent to the saltpan. While SI individuals experienced significantly greater water-stress than plants in II and MI plots, the Ψ_pd_ values were not low enough to generate the native embolism values measured in this study. These measurements were undertaken 2 years after the drought, so we assumed that the surviving individuals either refilled (Ewers et al., 2004; Schmitz et al., 2012) or regrew a new set of functional vessels in order to restore branch hydraulic conductivity. As embolism repair is unlikely under persistent water stress, embolism resulting from past stress events would have been preserved in the xylem. Therefore, the native PLC measured may represent a signal of the water stress experienced during die-off. We assumed that PLC measured 2 years after the event was lower than at the time of the mortality event because some level of conductivity would be recovered by growth of new xylem tissue in surviving branches. Based on this assumption, we speculated that mangroves reached values of embolism that exceeded 75% (i.e., highest native embolism measured in SI branches) during the extreme stress event. Studies investigating thresholds for drought induced mortality in woody plants indicate that water potentials causing 88% of loss of conductivity are a point of no-return for angiosperm species (Choat, 2013; Urli et al., 2013; Delzon and Cochard, 2014; Anderegg et al., 2015) although this threshold varies across species as some plants can reach over 90% of embolism and still recover (Hammond et al., 2019). *A. marina* survives higher stress than most plants due to high resistance to drought-induced embolism (Ψ_12_, Ψ_50_) and tolerance of high levels of embolism (Ψ_88_) before recovering. A higher resistance to drought-induced mortality would indicate that this species can endure extreme hydraulic stress.

In order to maintain a positive water balance, the leaf osmotic potential must be lower than the soil (Dixon and Joly, 1894). Despite this mechanism, some *A. marina* individuals in the seaward fringing plots experienced predawn water potentials higher than the water potential of the seawater (~−2.5 MPa). One explanation may be the existence of foliar water uptake during precipitation or when droplets of dew form on the leaves (Steppe et al., 2018). This phenomenon has been documented to improve leaf water status in a range of species and environments (Boucher et al., 1995; Yates and Hutley, 1995; Limm et al., 2009; Gerlein-Safdi et al., 2018). Mangrove species could also use foliar water uptake as a strategy to raise leaf water potential. The capacity of salt crystals located on the surface of the leaf to attract liquid and facilitate its entrance into the leaf has been discussed, although it seems unlikely due to the potential uptake of salty water through the stomata (Reef and Lovelock, 2015). However, *A. marina* is capable of taking up water from the leaf, which induces reverse sap flow through the plant and results in the restoration of favorable plant water balance and stem diameter growth (Steppe et al., 2018; Coopman et al., 2021). Leaf water uptake may also restore leaf hydraulic conductance and could help prevent hydraulic failure during drought (Fuenzalida et al., 2019; Coopman et al., 2021).

Mangroves growing adjacent to the saltpan were at a higher elevation (~1 m to 1.5 m) than those growing on the edge of the water (Sippo et al., 2020; Duke et al., 2021) and this difference in elevation may partly drive the difference in water availability along the transect. With the drop in sea level and lower rainfall observed during El Niño, it is possible that the soil water in SI areas was not sufficient to meet demand of transpiration in these trees (Xia and Li, 2012; Sippo et al., 2020). It is also likely that substrate salinity would increase in landward zone, given the absence of inundation and inputs of fresh water, placing further osmotic stress on mangroves (Lovelock et al., 2017). Differences in native embolism through this gradient suggest that water stress, was an important component of mortality during the 2015/16 El Niño event. With low sea level and low rainfall, the geochemistry of the sediment was also observed to change from reducing to oxidizing ions such as iron (Fe). Fe levels in mangrove wood were reported being 30 to 90 times higher in dead mangroves compared to baseline Fe (prior to dieback) (Sippo et al., 2020). Although *A. marina* may tolerate high concentrations of Fe (Johnston et al., 2016), it is likely that Fe toxicity also contributed to mangrove mortality during this event (Sippo et al., 2020).

## Conclusion

Mangrove species are found in estuaries and coastal shorelines of tropical and sub-tropical regions worldwide, where they face natural major disturbances associated with climate change and rising sea levels (Duke et al., 2007; Lovelock et al., 2017). However, recent mass dieback of mangroves in northern Australia has raised the Specter of a further profound impacts related to localized fluctuations in sea level (Duke et al., 2017, 2021; Sippo et al., 2020). In this study, it was notable that the die off event coincided with an intense El Niño event, and an unusually severe (~40 cm) and prolonged (over 6 months) localized drop in sea level (Duke et al., 2021). The combined impacts of reduced inundation with sea water, lower inputs of fresh water, and high VPD are likely to have resulted in an increase in substate salinity and imposition of severe water stress at many locations within the Gulf of Carpentaria. At our site, this was consistent with the pattern of impact, which was more severe for trees that were growing further from the seafront, suggesting that the degree of mortality was related to lower water availability. Physiological data demonstrates that trees in the SI zone are subject to higher background levels of water stress than those growing in the seaward zone, and that this stress was exacerbated during the die off event. The available evidence suggests a role for hydraulic failure if water potentials during the die off event dropped below thresholds causing catastrophic xylem embolism. This is supported by higher levels of native PLC in branches of surviving individuals in the SI plots, which may represent a signal of extreme water stress during the event. We acknowledge the possibility that higher native PLC in SI individuals could the result of background variation in water stress along an environmental gradient. Unfortunately, physiological data are not available from the period leading up to and during the die off event, making it difficult to draw unambiguous conclusions on this matter. However, the weight of evidence from remote sensing, analysis of relevant climatic processes (Duke et al., 2017; Abhik et al., 2021) and physiological data from the present study suggest that severe water stress was a major contributor to the die off event, with hydraulic failure strongly implicated as a mechanism of mortality. Further study is required to confirm these conclusions, with monitoring of sites likely to experience die off in the future a priority.

## Data Availability Statement

The raw data supporting the conclusions of this article will be made available by the authors, without undue reservation.

## Author Contributions

AG and BC designed the experiment with the help of the expertise of DM, LC, and ND. AG, JB, DB, IA, and DM carried out the fieldwork and helped perform the experiments. All authors contributed significantly to the writing of the manuscript.

## Funding

This study was supported by the Australia and Pacific Science Foundation and an ARC Future Fellowship to BC (FT130101115).

## Conflict of Interest

The authors declare that the research was conducted in the absence of any commercial or financial relationships that could be construed as a potential conflict of interest.

## Publisher's Note

All claims expressed in this article are solely those of the authors and do not necessarily represent those of their affiliated organizations, or those of the publisher, the editors and the reviewers. Any product that may be evaluated in this article, or claim that may be made by its manufacturer, is not guaranteed or endorsed by the publisher.
